# Supplementary material for: Intensity Paradox—Low-Fit People Are Physically Most Active in Terms of Their Fitness
Source: Sensors (Basel). 2021 Mar 15;21(6):2063. doi: 10.3390/s21062063 (PMC8002087; doi:10.3390/s21062063)
Supplement: Supplementary file 1 [file sensors-21-02063-s001.pdf]

Supplementary Materials

Intensity Paradox—Low-Fit People Are Physically Most Active in Terms of their Fitness

Henri Vähä-Ypyä [1], Harri Sievänen [1,*], Pauliina Husu [1], Kari Tokola [1] and Tommi Vasankari [1,2]

[1] UKK-Institute, 33500 Tampere, Finland; henri.vaha-ypya@ukkinstituutti.fi (H.V.-Y.); pauliina.husu@ukkinstituutti.fi (P.H.); kari.tokola@ukkinstituutti.fi (K.T.); tommi.vasankari@ukkinstituutti.fi (T.V.)

[2] Faculty of Medicine and Health Technology, Tampere University, 33014 Tampere, Finland

* Correspondence: harri.sievanen@ukkinstituutti.fi

Table S1. The table shows the parameter estimates of the multiple regression analysis for MVPA activity among men and women. The MVPA was analysed with absolute 3.0 MET threshold using both 6 s epochs and smoothed 1 and 6 min EMA. Natural logarithm of the accumulated MVPA time for different bout length was used as dependent variable. Age group (1 for 20–29; 2 for 30–39; 3 for 40–49; 4 for 50–59; 5 for 60–69) was used as a covariate and CRF third as a fixed factor.

		Women						Men					
		6 s epoch		1 min EMA		6 min EMA		6 s epoch		1 min EMA		6 min EMA	
**Bout**	**Variables**	**B**	**sig.**	**B**	**sig.**	**B**	**sig.**	**B**	**sig.**	**B**	**sig.**	**B**	**sig.**
≥ 0.1 min	Intercept	4.637	0.000	4.218	0.000	3.985	0.000	4.612	0.000	4.193	0.000	4.010	0.000
	Age group	−0.067	0.000	−0.086	0.000	−0.160	0.000	−0.067	0.000	−0.091	0.000	−0.168	0.000
	[Low CRF]	−0.281	0.000	−0.450	0.000	−0.842	0.000	−0.264	0.000	−0.429	0.000	−0.778	0.000
	[Middle CRF]	−0.093	0.010	−0.156	0.002	−0.311	0.007	−0.093	0.000	−0.162	0.000	−0.286	0.000
	[High CRF]	0[a]		0[a]		0[a]		0[a]		0[a]		0[a]	
≥ 0.5 min	Intercept	4.089	0.000	4.145	0.000	3.965	0.000	4.082	0.000	4.126	0.000	4.003	0.000
	Age group	−0.063	0.000	−0.087	0.000	−0.163	0.000	−0.070	0.000	−0.093	0.000	−0.171	0.000
	[Low CRF]	−0.360	0.000	−0.469	0.000	−0.830	0.000	−0.354	0.000	−0.449	0.000	−0.781	0.000
	[Middle CRF]	−0.119	0.010	−0.165	0.002	−0.295	0.013	−0.131	0.000	−0.172	0.000	−0.288	0.000
	[High CRF]	0[a]		0[a]		0[a]		0[a]		0[a]		0[a]	
≥ 1.0 min	Intercept	3.727	0.000	4.027	0.000	3.996	0.000	3.766	0.000	4.021	0.000	4.031	0.000
	Age group	−0.071	0.000	−0.095	0.000	−0.188	0.000	−0.079	0.000	−0.100	0.000	−0.194	0.000
	[Low CRF]	−0.440	0.000	−0.519	0.000	−0.863	0.000	−0.446	0.000	−0.505	0.000	−0.805	0.000
	[Middle CRF]	−0.152	0.013	−0.190	0.002	−0.282	0.037	−0.171	0.000	−0.194	0.000	−0.273	0.002
	[High CRF]	0[a]		0[a]		0[a]		0[a]		0[a]		0[a]	
≥ 3.0 min	Intercept	3.022	0.000	3.635	0.000	4.051	0.000	3.146	0.000	3.734	0.000	4.041	0.000
	Age group	−0.146	0.009	−0.156	0.001	−0.243	0.000	−0.145	0.000	−0.170	0.000	−0.229	0.000
	[Low CRF]	−0.830	0.000	−0.812	0.000	−1.150	0.000	−0.820	0.000	−0.758	0.000	−0.988	0.000
	[Middle CRF]	−0.174	0.321	−0.236	0.111	−0.363	0.041	−0.238	0.036	−0.278	0.003	−0.391	0.000
	[High CRF]	0[a]		0[a]		0[a]		0[a]		0[a]		0[a]	
≥ 5.0 min	Intercept	1.894	0.000	3.206	0.000	3.828	0.000	2.414	0.000	3.400	0.000	3.890	0.000
	Age group	−0.083	0.362	−0.190	0.007	−0.251	0.000	−0.146	0.006	−0.190	0.000	−0.243	0.000
	[Low CRF]	−1.334	0.000	−1.207	0.000	−1.343	0.000	−1.219	0.000	−1.098	0.000	−1.157	0.000
	[Middle CRF]	−0.410	0.152	−0.230	0.298	−0.344	0.108	−0.437	0.010	−0.419	0.002	−0.401	0.002
	[High CRF]	0[a]		0[a]		0[a]		0[a]		0[a]		0[a]	
≥ 10.0 min	Intercept	−0.392	0.442	1.899	0.000	3.515	0.000	0.389	0.206	2.409	0.000	3.683	0.000
	Age group	0.006	0.963	−0.186	0.086	−0.311	0.001	−0.076	0.303	−0.249	0.000	−0.329	0.000
	[Low CRF]	−1.400	0.000	−1.781	0.000	−2.054	0.000	−1.688	0.000	−1.712	0.000	−1.788	0.000
	[Middle CRF]	−0.806	0.034	−0.615	0.071	−0.746	0.012	−0.872	0.000	−0.694	0.001	−0.754	0.000
	[High CRF]	0[a]		0[a]		0[a]		0[a]		0[a]		0[a]	
≥ 20.0 min	Intercept	−3.514	0.000	−1.889	0.001	1.506	0.003	−2.918	0.000	−1.004	0.003	1.925	0.000
	Age group	0.130	0.283	0.192	0.136	−0.172	0.159	0.084	0.268	0.030	0.710	−0.272	0.000
	[Low CRF]	−1.250	0.001	−1.947	0.000	−2.344	0.000	−1.641	0.000	−1.927	0.000	−2.227	0.000
	[Middle CRF]	−0.508	0.182	−0.580	0.150	−1.083	0.005	−0.760	0.002	−0.608	0.018	−0.850	0.000

	[High CRF]	0[a]		0[a]		0[a]		0[a]		0[a]		0[a]	
≥ 30.0 min	Intercept	−4.948	0.000	−3.663	0.000	−0.469	0.398	−4.372	0.000	−2.917	0.000	0.122	0.720
	Age group	0.075	0.477	0.195	0.114	−0.066	0.617	0.040	0.540	0.102	0.187	−0.158	0.052
	[Low CRF]	−0.699	0.034	−1.630	0.000	−2.127	0.000	−1.363	0.000	−1.931	0.000	−2.428	0.000
	[Middle CRF]	−0.127	0.700	−0.395	0.308	−1.018	0.014	−0.648	0.002	−0.753	0.003	−1.054	0.000
	[High CRF]	0[a]		0[a]		0[a]		0[a]		0[a]		0[a]	
≥ 60.0 min	Intercept	−6.600	0.000	−6.029	0.000	−4.591	0.000	−6.469	0.000	−5.741	0.000	−4.602	0.000
	Age group	0.083	0.172	0.076	0.336	0.034	0.748	0.038	0.264	0.005	0.918	0.029	0.653
	[Low CRF]	−0.285	0.135	−0.646	0.010	−1.282	0.000	−0.351	0.001	−0.726	0.000	−1.338	0.000
	[Middle CRF]	0.078	0.683	0.027	0.913	−0.358	0.285	−0.073	0.507	−0.239	0.117	−0.511	0.014
	[High CRF]	0[a]		0[a]		0[a]		0[a]		0[a]		0[a]	

a This parameter is set to zero because it is redundant; EMA, exponential moving average; CRF cardiorespiratory fitness

Table S2. The table shows the parameter estimates of the multiple regression analysis for MVPA activity among men and women. The MVPA was analysed with relative 40% of VO2R threshold using both 6 s epochs and smoothed 1 and 6 min EMA. Natural logarithm of the accumulated MVPA time for different bout length was used as dependent variable. Age group (1 for 20–29; 2 for 30–39; 3 for 40–49; 4 for 50–59; 5 for 60–69) was used as a covariate and CRF third as a fixed factor.

		**Women**						**Men**					
		**6 s epoch**		**1 min EMA**		**6 min EMA**		**6 s epoch**		**1 min EMA**		**6 min EMA**	
**Bout**	**Variables**	**B**	**sig.**	**B**	**sig.**	**B**	**sig.**	**B**	**sig.**	**B**	**sig.**	**B**	**sig.**
≥ 0.1 min	Intercept	0.951	0.000	−3.339	0.000	0.951	0.000	1.769	0.000	0.871	0.000	−1.235	0.000
	Age group	0.257	0.000	0.536	0.000	0.257	0.000	0.150	0.000	0.171	0.000	0.267	0.000
	[Low CRF]	1.077	0.000	1.642	0.000	1.077	0.000	1.019	0.000	1.190	0.000	1.545	0.000
	[Middle CRF]	0.499	0.000	0.699	0.042	0.499	0.000	0.389	0.000	0.465	0.000	0.622	0.002
	[High CRF]	0[a]		0[a]		0[a]		0[a]		0[a]		0[a]	
≥ 0.5 min	Intercept	−0.705	0.007	−3.381	0.000	−0.705	0.007	0.767	0.000	0.543	0.001	−1.280	0.000
	Age group	0.375	0.000	0.538	0.000	0.375	0.000	0.200	0.000	0.195	0.000	0.268	0.000
	[Low CRF]	1.577	0.000	1.603	0.000	1.577	0.000	1.229	0.000	1.299	0.000	1.543	0.000
	[Middle CRF]	0.861	0.000	0.636	0.069	0.861	0.000	0.563	0.000	0.559	0.000	0.608	0.003
	[High CRF]	0[a]		0[a]		0[a]		0[a]		0[a]		0[a]	
≥ 1.0 min	Intercept	−2.370	0.000	−3.368	0.000	−2.370	0.000	−0.316	0.098	0.218	0.257	−1.377	0.000
	Age group	0.551	0.000	0.504	0.000	0.551	0.000	0.281	0.000	0.192	0.000	0.262	0.000
	[Low CRF]	1.846	0.000	1.587	0.000	1.846	0.000	1.449	0.000	1.401	0.000	1.577	0.000
	[Middle CRF]	1.051	0.000	0.595	0.096	1.051	0.000	0.729	0.000	0.603	0.000	0.586	0.005
	[High CRF]	0[a]		0[a]		0[a]		0[a]		0[a]		0[a]	
≥ 3.0 min	Intercept	−4.859	0.000	−3.874	0.000	−4.859	0.000	−2.423	0.000	−1.173	0.000	−1.871	0.000
	Age group	0.664	0.000	0.531	0.000	0.664	0.000	0.343	0.000	0.270	0.000	0.289	0.000
	[Low CRF]	2.073	0.000	1.429	0.000	2.073	0.000	1.850	0.000	1.519	0.000	1.544	0.000
	[Middle CRF]	1.164	0.001	0.547	0.146	1.164	0.001	0.988	0.000	0.753	0.000	0.595	0.008
	[High CRF]	0[a]		0[a]		0[a]		0[a]		0[a]		0[a]	
≥ 5.0 min	Intercept	−5.347	0.000	−4.325	0.000	−5.347	0.000	−3.435	0.000	−2.129	0.000	−2.219	0.000
	Age group	0.615	0.000	0.537	0.000	0.615	0.000	0.354	0.000	0.314	0.000	0.270	0.000
	[Low CRF]	1.386	0.000	1.305	0.001	1.386	0.000	1.617	0.000	1.452	0.000	1.496	0.000
	[Middle CRF]	0.860	0.023	0.649	0.094	0.860	0.023	0.745	0.002	0.648	0.004	0.614	0.009
	[High CRF]	0[a]		0[a]		0[a]		0[a]		0[a]		0[a]	
≥ 10.0 min	Intercept	−5.741	0.000	−4.819	0.000	−5.741	0.000	−4.588	0.000	−3.613	0.000	−3.088	0.000
	Age group	0.396	0.001	0.515	0.000	0.396	0.001	0.258	0.001	0.305	0.000	0.295	0.000
	[Low CRF]	0.711	0.051	0.621	0.116	0.711	0.051	0.892	0.000	1.048	0.000	1.073	0.000
	[Middle CRF]	0.487	0.182	0.283	0.475	0.487	0.182	0.344	0.156	0.328	0.192	0.240	0.344
	[High CRF]	0[a]		0[a]		0[a]		0[a]		0[a]		0[a]	
≥ 20.0 min	Intercept	−5.872	0.000	−5.784	0.000	−5.872	0.000	−5.830	0.000	−4.978	0.000	−4.472	0.000
	Age group	0.173	0.074	0.472	0.000	0.173	0.074	0.185	0.003	0.265	0.000	0.322	0.000
	[Low CRF]	−0.073	0.810	0.341	0.366	−0.073	0.810	0.112	0.577	0.124	0.599	0.538	0.034
	[Middle CRF]	0.009	0.977	0.300	0.427	0.009	0.977	0.091	0.651	−0.063	0.791	0.124	0.626

	[High CRF]	0[a]		0[a]		0[a]		0[a]		0[a]		0[a]	
≥ 30.0 min	Intercept	−6.043	0.000	−5.994	0.000	−6.043	0.000	−6.017	0.000	−5.794	0.000	−5.022	0.000
	Age group	0.055	0.486	0.336	0.002	0.055	0.486	0.041	0.403	0.183	0.003	0.196	0.007
	[Low CRF]	−0.271	0.272	0.066	0.849	−0.271	0.272	−0.115	0.463	−0.085	0.671	0.192	0.413
	[Middle CRF]	0.020	0.937	0.097	0.780	0.020	0.937	−0.080	0.610	−0.047	0.813	−0.123	0.599
	[High CRF]	0[a]		0[a]		0[a]		0[a]		0[a]		0[a]	
≥ 60.0 min	Intercept	−6.535	0.000	−6.478	0.000	−6.535	0.000	−6.580	0.000	−6.473	0.000	−6.382	0.000
	Age group	−0.035	0.367	0.073	0.280	−0.035	0.367	−0.031	0.150	0.014	0.676	0.051	0.239
	[Low CRF]	−0.106	0.383	−0.182	0.390	−0.106	0.383	−0.101	0.147	−0.161	0.124	−0.032	0.818
	[Middle CRF]	0.005	0.967	0.056	0.793	0.005	0.967	−0.042	0.547	−0.126	0.230	−0.014	0.917
	[High CRF]	0[a]		0[a]		0[a]		0[a]		0[a]		0[a]	

a This parameter is set to zero because it is redundant; EMA, exponential moving average; CRF cardiorespiratory fitness.

Table S3. The table shows the parameter estimates of the multiple regression analysis for VPA activity among men and women. The VPA was analysed with absolute 6.0 MET threshold using both 6 s epochs and smoothed 1 and 6 min EMA. Natural logarithm of the accumulated VPA time for different bout length was used as dependent variable. Age group (1 for 20–29; 2 for 30–39; 3 for 40–49; 4 for 50–59; 5 for 60–69) was used as a covariate and CRF third as a fixed factor.

		Women						Men					
		6 s epoch		1 min EMA		6 min EMA		6 s epoch		1 min EMA		6 min EMA	
**Bout**	**Variables**	**B**	**sig.**	**B**	**sig.**	**B**	**sig.**	**B**	**sig.**	**B**	**sig.**	**B**	**sig.**
≥ 0.1 min	Intercept	2.734	0.000	1.901	0.000	−0.678	0.103	2.844	0.000	2.555	0.000	−0.285	0.251
	Age group	−0.618	0.000	−0.892	0.000	−0.720	0.000	−0.665	0.000	−1.062	0.000	−0.828	0.000
	[Low CRF]	−1.884	0.000	−3.047	0.000	−2.496	0.000	−1.875	0.000	−3.080	0.000	−2.557	0.000
	[Middle CRF]	−0.798	0.000	−1.401	0.000	−1.168	0.000	−0.884	0.000	−1.482	0.000	−1.431	0.000
	[High CRF]	0[a]		0[a]		0[a]		0[a]		0[a]		0[a]	
≥ 0.5 min	Intercept	1.625	0.000	1.359	0.001	−0.783	0.060	2.327	0.000	2.162	0.000	−0.352	0.157
	Age group	−0.909	0.000	−0.898	0.000	−0.701	0.000	−1.089	0.000	−1.095	0.000	−0.824	0.000
	[Low CRF]	−3.255	0.000	−3.034	0.000	−2.479	0.000	−3.156	0.000	−3.031	0.000	−2.515	0.000
	[Middle CRF]	−1.526	0.000	−1.321	0.000	−1.242	0.000	−1.568	0.000	−1.465	0.000	−1.450	0.000
	[High CRF]	0[a]		0[a]		0[a]		0[a]		0[a]		0[a]	
≥ 1.0 min	Intercept	0.102	0.802	0.574	0.175	−0.887	0.033	0.852	0.001	1.449	0.000	−0.459	0.064
	Age group	−0.756	0.000	−0.797	0.000	−0.690	0.000	−0.961	0.000	−1.010	0.000	−0.817	0.000
	[Low CRF]	−3.010	0.000	−3.111	0.000	−2.436	0.000	−2.856	0.000	−3.075	0.000	−2.441	0.000
	[Middle CRF]	−1.496	0.000	−1.344	0.000	−1.268	0.000	−1.518	0.000	−1.519	0.000	−1.464	0.000
	[High CRF]	0[a]		0[a]		0[a]		0[a]		0[a]		0[a]	
≥ 3.0 min	Intercept	−2.310	0.000	−1.378	0.001	−1.518	0.000	−1.818	0.000	−0.472	0.058	−0.965	0.000
	Age group	−0.531	0.000	−0.616	0.000	−0.640	0.000	−0.658	0.000	−0.813	0.000	−0.766	0.000
	[Low CRF]	−1.864	0.000	−2.319	0.000	−2.092	0.000	−1.925	0.000	−2.440	0.000	−2.227	0.000
	[Middle CRF]	−0.972	0.001	−1.205	0.000	−1.182	0.000	−1.137	0.000	−1.377	0.000	−1.397	0.000
	[High CRF]	0[a]		0[a]		0[a]		0[a]		0[a]		0[a]	
≥ 5.0 min	Intercept	−3.162	0.000	−2.063	0.000	−1.924	0.000	−2.977	0.000	−1.682	0.000	−1.457	0.000
	Age group	−0.438	0.000	−0.564	0.000	−0.577	0.000	−0.541	0.000	−0.663	0.000	−0.713	0.000
	[Low CRF]	−1.611	0.000	−2.022	0.000	−2.077	0.000	−1.404	0.000	−2.013	0.000	−2.057	0.000
	[Middle CRF]	−0.792	0.003	−1.023	0.001	−1.196	0.000	−0.767	0.000	−1.187	0.000	−1.286	0.000
	[High CRF]	0[a]		0[a]		0[a]		0[a]		0[a]		0[a]	
≥ 10.0 min	Intercept	−3.818	0.000	−3.280	0.000	−2.937	0.000	−4.325	0.000	−3.333	0.000	−2.730	0.000
	Age group	−0.365	0.000	−0.393	0.000	−0.456	0.000	−0.331	0.000	−0.450	0.000	−0.544	0.000
	[Low CRF]	−1.445	0.000	−1.711	0.000	−1.634	0.000	−1.087	0.000	−1.463	0.000	−1.602	0.000
	[Middle CRF]	−0.856	0.000	−1.003	0.000	−0.936	0.001	−0.616	0.000	−0.902	0.000	−1.004	0.000
	[High CRF]	0[a]		0[a]		0[a]		0[a]		0[a]		0[a]	
≥ 20.0 min	Intercept	−4.783	0.000	−4.030	0.000	−3.604	0.000	−5.242	0.000	−4.467	0.000	−3.871	0.000
	Age group	−0.278	0.000	−0.342	0.000	−0.360	0.000	−0.235	0.000	−0.302	0.000	−0.370	0.000
	[Low CRF]	−0.920	0.000	−1.295	0.000	−1.555	0.000	−0.649	0.000	−1.089	0.000	−1.333	0.000
	[Middle CRF]	−0.636	0.001	−0.912	0.000	−0.996	0.000	−0.407	0.000	−0.682	0.000	−0.803	0.000

	[High CRF]	0[a]		0[a]		0[a]		0[a]		0[a]		0[a]	
≥ 30.0 min	Intercept	−5.621	0.000	−5.121	0.000	−4.347	0.000	−5.962	0.000	−5.460	0.000	−4.788	0.000
	Age group	−0.168	0.001	−0.192	0.002	−0.289	0.000	−0.124	0.000	−0.164	0.000	−0.241	0.000
	[Low CRF]	−0.617	0.000	−0.850	0.000	−1.131	0.000	−0.454	0.000	−0.728	0.000	−1.040	0.000
	[Middle CRF]	−0.300	0.056	−0.566	0.005	−0.713	0.003	−0.200	0.025	−0.458	0.000	−0.669	0.000
	[High CRF]	0[a]		0[a]		0[a]		0[a]		0[a]		0[a]	
≥ 60.0 min	Intercept	−6.487	0.000	−6.329	0.000	−5.945	0.000	−6.675	0.000	−6.519	0.000	−6.168	0.000
	Age group	−0.048	0.112	−0.073	0.042	−0.128	0.008	−0.025	0.099	−0.045	0.020	−0.090	0.000
	[Low CRF]	−0.212	0.026	−0.247	0.028	−0.321	0.033	−0.115	0.019	−0.187	0.002	−0.319	0.000
	[Middle CRF]	−0.138	0.148	−0.167	0.137	−0.210	0.163	−0.071	0.147	−0.112	0.070	−0.231	0.005
	[High CRF]	0[a]		0[a]		0[a]		0[a]		0[a]		0[a]	

a This parameter is set to zero because it is redundant; EMA, exponential moving average; CRF cardiorespiratory fitness.

Table S4. The table shows the parameter estimates of the multiple regression analysis for VPA activity among men and women. The MVPA was analysed with relative 40% of VO2R threshold using both 6 s epochs and smoothed 1 and 6 min EMA. Natural logarithm of the accumulated MVPA time for different bout length was used as dependent variable. Age group (1 for 20–29; 2 for 30–39; 3 for 40–49; 4 for 50–59; 5 for 60–69) was used as a covariate and CRF third as a fixed factor.

		**Women**						**Men**					
		**6 s epoch**		**1 min EMA**		**6 min EMA**		**6 s epoch**		**1 min EMA**		**6 min EMA**	
**Bout**	**Variables**	**B**	**sig.**	**B**	**sig.**	**B**	**sig.**	**B**	**sig.**	**B**	**sig.**	**B**	**sig.**
≥ 0.1 min	Intercept	−0.662	0.007	−2.772	0.000	−4.097	0.000	−0.662	0.007	−2.772	0.000	−4.097	0.000
	Age group	−0.012	0.835	−0.086	0.400	−0.157	0.125	−0.012	0.835	−0.086	0.400	−0.157	0.125
	[Low CRF]	0.979	0.000	1.144	0.000	0.295	0.355	0.979	0.000	1.144	0.000	0.295	0.355
	[Middle CRF]	−0.073	0.689	−0.287	0.371	−0.420	0.189	−0.073	0.689	−0.287	0.371	−0.420	0.189
	[High CRF]	0[a]		0[a]		0[a]		0[a]		0[a]		0[a]	
≥ 0.5 min	Intercept	−3.336	0.000	−3.271	0.000	−4.116	0.000	−3.336	0.000	−3.271	0.000	−4.116	0.000
	Age group	−0.002	0.987	−0.088	0.412	−0.159	0.119	−0.002	0.987	−0.088	0.412	−0.159	0.119
	[Low CRF]	1.092	0.001	1.119	0.001	0.287	0.368	1.092	0.001	1.119	0.001	0.287	0.368
	[Middle CRF]	−0.457	0.162	−0.283	0.398	−0.436	0.172	−0.457	0.162	−0.283	0.398	−0.436	0.172
	[High CRF]	0[a]		0[a]		0[a]		0[a]		0[a]		0[a]	
≥ 1.0 min	Intercept	−4.231	0.000	−3.883	0.000	−4.212	0.000	−4.231	0.000	−3.883	0.000	−4.212	0.000
	Age group	−0.018	0.863	−0.031	0.773	−0.150	0.139	−0.018	0.863	−0.031	0.773	−0.150	0.139
	[Low CRF]	0.991	0.002	0.842	0.013	0.280	0.378	0.991	0.002	0.842	0.013	0.280	0.378
	[Middle CRF]	−0.416	0.202	−0.297	0.382	−0.431	0.176	−0.416	0.202	−0.297	0.382	−0.431	0.176
	[High CRF]	0[a]		0[a]		0[a]		0[a]		0[a]		0[a]	
≥ 3.0 min	Intercept	−4.306	0.000	−4.545	0.000	−4.206	0.000	−4.306	0.000	−4.545	0.000	−4.206	0.000
	Age group	−0.216	0.020	−0.076	0.454	−0.199	0.045	−0.216	0.020	−0.076	0.454	−0.199	0.045
	[Low CRF]	0.163	0.576	0.530	0.098	0.229	0.460	0.163	0.576	0.530	0.098	0.229	0.460
	[Middle CRF]	−0.551	0.059	−0.376	0.241	−0.445	0.152	−0.551	0.059	−0.376	0.241	−0.445	0.152
	[High CRF]	0[a]		0[a]		0[a]		0[a]		0[a]		0[a]	
≥ 5.0 min	Intercept	−4.548	0.000	−4.606	0.000	−4.387	0.000	−4.548	0.000	−4.606	0.000	−4.387	0.000
	Age group	−0.236	0.005	−0.136	0.158	−0.192	0.046	−0.236	0.005	−0.136	0.158	−0.192	0.046
	[Low CRF]	−0.191	0.473	0.207	0.492	−0.061	0.838	−0.191	0.473	0.207	0.492	−0.061	0.838
	[Middle CRF]	−0.471	0.077	−0.411	0.173	−0.379	0.208	−0.471	0.077	−0.411	0.173	−0.379	0.208
	[High CRF]	0[a]		0[a]		0[a]		0[a]		0[a]		0[a]	
≥ 10.0 min	Intercept	−4.866	0.000	−4.573	0.000	−4.294	0.000	−4.866	0.000	−4.573	0.000	−4.294	0.000
	Age group	−0.265	0.000	−0.222	0.010	−0.260	0.004	−0.265	0.000	−0.222	0.010	−0.260	0.004
	[Low CRF]	−0.307	0.169	−0.309	0.249	−0.388	0.166	−0.307	0.169	−0.309	0.249	−0.388	0.166
	[Middle CRF]	−0.396	0.075	−0.554	0.039	−0.517	0.066	−0.396	0.075	−0.554	0.039	−0.517	0.066
	[High CRF]	0[a]		0[a]		0[a]		0[a]		0[a]		0[a]	
≥ 20.0 min	Intercept	−5.709	0.000	−4.982	0.000	−4.729	0.000	−5.709	0.000	−4.982	0.000	−4.729	0.000
	Age group	−0.141	0.008	−0.232	0.001	−0.220	0.006	−0.141	0.008	−0.232	0.001	−0.220	0.006
	[Low CRF]	−0.431	0.010	−0.551	0.009	−0.536	0.033	−0.431	0.010	−0.551	0.009	−0.536	0.033
	[Middle CRF]	−0.313	0.062	−0.566	0.007	−0.483	0.055	−0.313	0.062	−0.566	0.007	−0.483	0.055

	[High CRF]	0[a]		0[a]		0[a]		0[a]		0[a]		0[a]	
≥ 30.0 min	Intercept	−6.165	0.000	−5.745	0.000	−5.487	0.000	−6.165	0.000	−5.745	0.000	−5.487	0.000
	Age group	−0.092	0.028	−0.153	0.005	−0.145	0.036	−0.092	0.028	−0.153	0.005	−0.145	0.036
	[Low CRF]	−0.316	0.015	−0.309	0.070	−0.368	0.090	−0.316	0.015	−0.309	0.070	−0.368	0.090
	[Middle CRF]	−0.163	0.211	−0.196	0.251	−0.133	0.539	−0.163	0.211	−0.196	0.251	−0.133	0.539
	[High CRF]	0[a]		0[a]		0[a]		0[a]		0[a]		0[a]	
≥ 60.0 min	Intercept	−6.592	0.000	−6.530	0.000	−6.410	0.000	−6.592	0.000	−6.530	0.000	−6.410	0.000
	Age group	−0.039	0.118	−0.056	0.073	−0.059	0.134	−0.039	0.118	−0.056	0.073	−0.059	0.134
	[Low CRF]	−0.177	0.022	−0.108	0.265	−0.106	0.390	−0.177	0.022	−0.108	0.265	−0.106	0.390
	[Middle CRF]	−0.104	0.181	−0.029	0.764	−0.098	0.429	−0.104	0.181	−0.029	0.764	−0.098	0.429
	[High CRF]	0[a]		0[a]		0[a]		0[a]		0[a]		0[a]	

a This parameter is set to zero because it is redundant; EMA, exponential moving average; CRF cardiorespiratory fitness.
